# Sympathetic Stress and Sleep Loss in Diabetic Retinopathy: Links to Retinal Blood-Flow Control

**DOI:** 10.3390/biomedicines14030736

**Published:** 2026-03-23

**Authors:** Mengquan Tan, Shengtao Liu, Muxuan Fang, Man Yuan, Danping Niu, Yang Wang, Huixian Zhou, Jiling Zeng, Yaling Dai, Siyuan Song

**Affiliations:** 1 Institute of Rehabilitation Industry, Fujian University of Traditional Chinese Medicine, Fuzhou 350122, China; 2 Department of Traditional Chinese Medicine Rehabilitation, The First People’s Hospital of Dazhou City, Dazhou 635000, China; 3 Fujian Key Laboratory of Aptamers Technology, Fuzhou General Teaching Hospital (The 900thHospital), Fujian University of Traditional Chinese Medicine, Fuzhou 350025, China; 4 Eye Center, Renmin Hospital of Wuhan University, Wuhan University, Wuhan 430060, China; 5 State Key Laboratory of Virology, College of Life Sciences, Wuhan University, Wuhan 430060, China; 6 Intensive Care Unit, The 960th Hospital of the Chinese People’s Liberation Army Joint Logistics Support Force, Jinan 250031, China; 7 Department of Internal Medicine, Montefiore Medical Center, Albert Einstein College of Medicine, New York, NY 10461, USA; 8 Sun Yat-sen University Cancer Center, Guangzhou 510060, China; zengjiling1001@gmail.com; 9 Department of Neuroscience, Baylor College of Medicine, Houston, TX 77030, USA

**Keywords:** diabetic retinopathy, neurovascular unit, neurovascular coupling, sympathetic activation, sleep deprivation, neuroinflammation, pericytes, perioperative stress

## Abstract

Diabetic retinopathy (DR) is more than a capillary disorder. Diabetes affects neurons, glial cells, vascular cells, and immune signals within the retinal neurovascular unit (NVU). Retinal neurovascular coupling (NVC) is a useful functional marker of NVU integrity because it reflects the rise in local blood flow that follows neural activity. Many human flicker-light studies report smaller vessel dilation or weaker flow responses in diabetes. This finding can appear even in patients without clear fundus lesions. When NVC is reduced, retinal tissue may receive less oxygen. Lower oxygen delivery can raise oxidative stress and promote inflammation. These changes can then worsen vascular injury. This review describes key NVC pathways and diabetes-related NVU changes in Müller glia, astrocytes, microglia, pericytes, and endothelial cells. The review highlights sympathetic activation as a common stress signal. Pain, anxiety, perioperative stress, and sleep loss can increase sympathetic activity and circulating catecholamines. In the diabetic retina, vascular reserve is often limited. Under these conditions, catecholamines can increase mural cell constriction, reduce nitric oxide (NO)-dependent relaxation, and increase endothelial activation and barrier strain. These effects can shift the baseline state of glial and immune cells and further weaken NVC. The review also summarizes translational tools that can test these links. These tools include heart rate variability, standardized NVC protocols with diameter and flow measures, and retinal organoid and organ-on-a-chip platforms with controlled adrenergic exposure. The review discusses perioperative care packages that reduce stress responses, protect sleep, and manage glucose as practical ways to support retinal microcirculation. More longitudinal human studies are still needed. Retina-specific perioperative endpoints are also needed to clarify causality and to guide intervention trials.

## 1. Introduction

Diabetic retinopathy (DR) is often described as a microvascular complication of diabetes [1]. This view does not explain several early changes in the diabetic retina [2]. Many studies now describe DR as a disorder of the retinal neurovascular unit (NVU) [2,3,4]. The NVU includes neurons, Müller glia, astrocytes, microglia, pericytes, endothelial cells, and the extracellular matrix [3,5]. Diabetes can disturb these cells at the same time, not in a strict sequence [1]. This NVU model also fits clinical data showing that neural dysfunction and glial activation can appear before obvious microvascular lesions on fundus examination [2,6,7,8].

Retinal neurovascular coupling (NVC), also called functional hyperemia, is a practical readout of NVU integrity [9,10,11]. NVC links neural activity to local increases in blood flow [9]. The healthy retina uses this response to match oxygen and nutrient delivery to moment-to-moment metabolic demand [9,10]. Neurons provide the initiating signals [10,12]. Müller glia and astrocytes shape the response through calcium-dependent pathways and by releasing vasoactive mediators [12,13,14]. Endothelial nitric oxide (NO) signaling and pericyte–vascular smooth muscle control then set the final vessel tone [1,9]. Experimental work supports a central role for glial signaling in driving activity-evoked vasodilation in the retina [10,13,14].

Human studies can measure NVC with flicker-light stimulation and vessel diameter or flow readouts [9,10,15,16]. Dynamic vessel analysis and related approaches often detect a reduced flicker-evoked dilation in diabetes, including in some patients without advanced visible DR [17,18,19,20,21]. These findings support the idea that NVC can fail early and can reflect “reserve” loss in vascular control [17,18,20,21,22]. Newer imaging, including functional OCT angiography (fOCTA), can map capillary-level hyperemia and may improve spatial specificity for translation and trials [23,24,25].

Diabetes makes NVC unreliable [9]. Diabetes reduces NO bioavailability, increases oxidative stress, and weakens endothelial barrier function [26,27,28,29]. Diabetes also changes glial reactivity and microglial immune signaling [3,5,30]. When activity-evoked blood flow is blunted, neural demand and vascular supply separate [9,10]. This mismatch can increase local hypoxia and metabolic stress [9,10,31,32]. The same stress can then amplify inflammation, blood–retinal barrier (BRB) dysfunction, and capillary nonperfusion [28,29,31,32,33]. Vascular injury can then worsen neural and glial dysfunction and further weaken coupling [3,5,9,31]. Reviews and mechanistic studies support this self-reinforcing NVU loop in DR [3,5,31,34].

This review focuses on sympathetic activation as a state-dependent amplifier of NVU vulnerability in diabetes [35]. Diabetes lowers vascular reserve and weakens barrier and glial homeostasis. A sympathetic surge can add adrenergic drive and endothelial stress on this baseline. This interaction may worsen NVC and increase inflammatory instability.

Pain, anxiety, surgery, and sleep loss can increase sympathetic outflow and circulating catecholamines [35,36,37,38]. These responses can bias microvessels toward constriction and can disrupt endothelial signaling [39,40]. These responses can also shift innate immune tone [41]. Retinal cells express adrenergic receptors, and retinal vessels can become more sensitive to adrenergic constrictor signaling when endothelial function is impaired [42,43]. This pattern is relevant to diabetes, because diabetes already limits vascular reserve [44,45]. Recent work also links hyperglycemia to increased circulating norepinephrine that enters the retina and alters microglial behavior through β2-adrenergic signaling, with downstream effects on vascular repair and barrier stability [46].

Sleep disturbance adds a second clinically relevant route [38]. Meta-analyses link sleep problems and obstructive sleep apnea (OSA) with higher DR risk or worse DR outcomes in many cohorts [47,48,49]. OSA also increases sympathetic tone and exposes tissues to intermittent hypoxia, which can stress endothelial and immune pathways that also support NVC [38,50,51,52]. These links are actionable in perioperative and pain medicine, because clinicians can often measure autonomic state and can modify sleep and stress responses [6,36,45].

In this review, we summarize the core mechanisms that generate retinal NVC in the NVU. We describe how diabetes disrupts each NVU component and how these disruptions converge on NVC failure. We next focus on adrenergic and neuroimmune pathways that may connect sympathetic activation, pain-related stress, perioperative stress, and sleep loss to worse NVC and NVU instability. Finally, we outline translational strategies that can test these ideas, including autonomic markers (for example, heart rate variability and catecholamine-linked surrogates), standardized human NVC testing (flicker protocols with flow or diameter readouts), and model systems that allow direct manipulation of adrenergic and microglial signaling.

## 2. Retinal Neurovascular Unit and Neurovascular Coupling

### 2.1. Cellular Architecture of the Retinal NVU

The retina has a high energy demand and low tolerance for supply failure [53]. The retina therefore relies on NVC to match local blood flow to local neural activity [10,12]. The retinal NVU supports this process [12]. The NVU works as a linked signaling network, not as a single structure [12]. Each NVU cell receives activity or stress signals, converts them into molecular outputs, and passes them to other NVU cells. These linked steps finally change vessel tone, flow distribution, and barrier permeability [12,54].

Neural activity provides the initiating physiological inputs for NVC [10]. Retinal neurons generate this signal during visual processing [10]. Photoreceptors, bipolar cells, amacrine cells, and retinal ganglion cells change synaptic transmission and metabolic use [10]. Neuronal activity increases glutamate release and shifts extracellular potassium in the inner retina [10]. Neurons also send vasoactive cues directly. Neuronal nitric oxide synthase produces NO, and NO can relax mural cells through cGMP signaling [10,55]. Neurons also change oxygen consumption and lactate production, and these metabolic shifts can shape the local extracellular environment. These neuron-derived signals create the input that the rest of the NVU must interpret and convert into a vascular response [10,12].

Müller glia mediate activity-dependent signaling between synaptic circuits and the microvasculature [14]. Müller glia span the entire retinal thickness, so Müller cells can link synapses to vessels across layers. Müller cells sense glutamate through metabotropic glutamate receptors and sense ATP through purinergic receptors [14,56]. These inputs drive intracellular calcium signaling in Müller processes near synapses and near vessel walls [13,14]. Calcium-dependent pathways then control mediator release [13,14,57]. Müller cells can produce prostaglandins through cyclooxygenase pathways and can shape epoxygenase products such as EETs [14,57]. These mediators act on mural cells and change tone [57]. Müller cells also stabilize the perivascular milieu through Kir4.1 and AQP4, which control potassium and water near vessels [58]. When these homeostatic functions weaken, the local vascular response becomes less stable, and NVC becomes less reliable [13,14].

Astrocytes add a second glial layer that is especially important for the superficial circulation [59]. Astrocyte endfeet contact vessels in the nerve fiber and ganglion cell layers [59]. Astrocytes detect activity-linked signals and ATP, and astrocytes generate calcium responses [10,59]. Astrocytes then release vasoactive factors that influence arterioles [59]. Astrocytes also support barrier maintenance through trophic signals and matrix-related cues that stabilize endothelial junctions and the basement membrane [54,59]. These astrocyte functions can keep the superficial vascular tree responsive, and they can keep barrier control aligned with local neural demand [59].

Immune signaling then shapes the baseline state in which NVC operates [11,12,60]. Microglia provide surveillance, but microglia also act as active NVU regulators [60]. Microglia sense ATP and damage signals through purinergic and innate immune pathways [60,61]. Microglia can release cytokines such as IL-1β and TNF-α, and these cytokines act on Müller glia and endothelial cells [60,61,62]. This shift can move the NVU toward an inflammatory set point [62]. This set point can increase tone variability and can reduce signal fidelity, so the same neural input triggers a weaker or noisier vascular output [11]. In diabetes, this immune bias can appear early, so NVC can fail even before prominent structural lesions are present [11,60].

Vascular cells execute the final response and enforce the inner blood–retina barrier [12,54]. Endothelial cells integrate shear stress and chemical cues and produce NO through Enos [54]. Endothelial tight junction complexes that include claudin-5, occludin, and ZO-1 support barrier integrity [54]. Under inflammatory conditions, endothelial cells increase adhesion molecules such as ICAM-1, and this change promotes leukocyte adhesion and capillary flow disturbance [54]. Pericytes provide fine capillary control, while smooth muscle cells control larger arterioles [63]. Both mural cell types convert vasoactive inputs into calcium–myosin signals that prompt change diameter [63]. This arrangement explains why NVC is fragile [10,12]. A neuron can signal demand, but NVC still fails if glial mediator release is distorted, if endothelial NO signaling is impaired, or if mural cells cannot respond.

**Figure 1:**
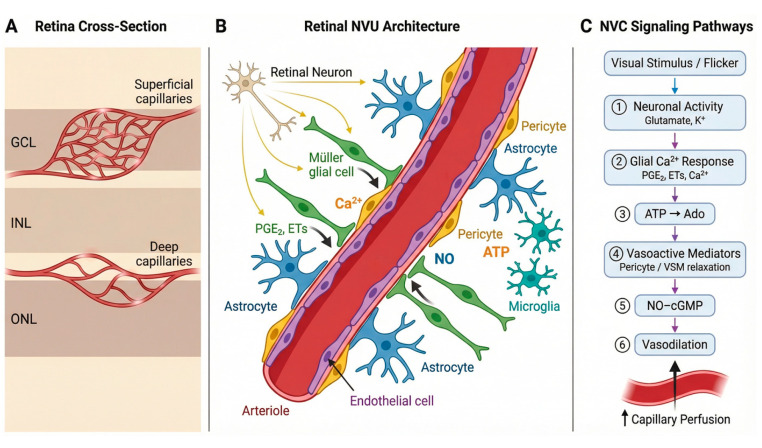
NVU organization and the core NVC pathway. (**A**) This panel shows a simplified retinal cross-section. The schematic highlights the superficial and deep capillary plexuses across key retinal layers, including the ganglion cell layer (GCL), inner nuclear layer (INL), and outer nuclear layer (ONL). (**B**) This panel summarizes the cellular layout of the retinal NVU around the microvasculature. Retinal neurons provide the initiating activity signals by increasing glutamate release and shifting extracellular K^+^. Müller glia and astrocytes detect these signals and generate intracellular Ca^2+^ responses. These glial Ca^2+^ signals drive the release of vasoactive mediators, including prostaglandin E2 (PGE2) and epoxyeicosatrienoic acids (EETs). Extracellular ATP contributes to signaling and can be converted to adenosine (Ado). Endothelial cells contribute additional vascular control by producing nitric oxide (NO), which influences mural cell tone. Microglia provide immune-related inputs that can shift the baseline state in which NVC operates. (**C**) This panel presents a stepwise view of flicker-evoked NVC. Visual stimulation increases neuronal activity, which then triggers glial Ca^2+^ signaling and mediator release. These signals converge on pericytes and vascular smooth muscle cells, in part through NO-cGMP signaling. This convergence relaxes mural cells, causes vasodilation, and increases capillary perfusion (functional hyperemia). Arrows indicate the direction of signaling, communication, or response pathways.

This NVU framework emphasizes early functional impairment [11]. NVC depends on coordinated signaling across neurons, glia, immune cells, and vascular cells. Modest dysfunction in any component can reduce the magnitude and reliability of activity-linked hyperemia [12]. Diabetes can disturb neuronal excitability, glial reactivity, immune signaling, and endothelial function in parallel [60]. These changes reduce vascular reserve and weaken the precision of NVU regulation. This logic supports NVC as an early functional indicator of NVU integrity in diabetic retina (Figure 1) [11].

### 2.2. Molecular Logic of Retinal Functional Hyperemia

NVC depends on a sequence of signaling events that link neural activity to local perfusion (Figure 1). Neuronal activity changes extracellular transmitters, ions, and metabolites. Glial and vascular cells detect these changes through specific receptors. These cells then regulate mediator pathways that act on mural cells and endothelial cells. The final output is a change in vessel diameter and a change in capillary perfusion pattern [10,12].

Visual stimulation increases neural activity and local metabolic demand [10,12]. Neurons release glutamate and change extracellular K^+^, and glial cells detect these signals and generate Ca^2+^-dependent responses [10,12,13]. These steps drive mediator release that acts on mural cells and endothelium. NO and arachidonic-acid-linked lipid mediators provide two major effector systems for dilation, while adenosine and K^+^ can modulate tone under some conditions [10,12,64,65,66,67]. Sympathetic activation can shift this balance by raising baseline constrictor tone and by weakening NO buffering when oxidative stress is high [42,43,68].

Pericytes and vascular smooth muscle cells change intracellular calcium and myosin phosphorylation in response to vasoactive mediators [69,70]. Endothelial cells also contribute by coordinating dilation signals and by regulating barrier properties [26,69]. Pericyte–endothelial connectivity supports spatially organized responses in the capillary bed [69]. Diabetes disrupts this connectivity map and can reduce the precision of local perfusion control [69,70].

These pathway dependencies explain early NVC impairment in diabetes [2,5]. Diabetes reduces NO bioavailability and increases oxidative stress [2,26]. Diabetes also shifts glial signaling and inflammatory tone [2,5]. Diabetes can reduce mural cell responsiveness and can weaken capillary-level coordination [2,70]. Clinical studies show reduced flicker-evoked retinal vasodilation in diabetes, and reviews summarize consistent evidence for impaired NVC in early disease [2,71,72]. New imaging approaches also detect altered NVC across macular plexuses and at capillary resolution before advanced clinical DR in some cohorts [25,71].

### 2.3. Pericytes and Gap Junction Signaling in Retinal Vascular Control

Pericytes cover most retinal capillaries [73]. They contribute to capillary diameter regulation and local flow distribution [74]. Pericytes change capillary diameter through contractile machinery that depends on intracellular calcium [63,75]. Nitric oxide can reduce pericyte contractile tone through the sGC–cGMP pathway, and this change supports dilation under conditions that preserve nitric oxide bioavailability [76]. Lipid mediators, including prostaglandins, can also modulate pericyte tone through receptor-linked signaling [77]. In diabetes, oxidative stress and inflammatory mediators can increase calcium-dependent constrictor responses and can reduce the dynamic range of pericyte vasomotor behavior [78,79]. Experimental work in the mouse retina shows that diabetes weakens pericyte-driven vasomotor responses during early disease stages [70].

Capillary regulation also depends on coordinated signaling along the vascular wall [69]. Endothelial cells can spread electrical and chemical signals along capillaries, and pericytes can couple to this process through direct cell–cell communication [80]. Gap junction channels formed by connexins permit intercellular transfer of ions and small signaling molecules, which supports coordinated diameter control across connected capillary segments [80]. In the mouse retinal microvasculature, studies that combine tracer coupling and connexin mapping show structured patterns of vascular gap junction coupling, including prominent roles for connexins such as Connexin43 [69,70].

Multiple lines of evidence link diabetes to impaired Connexin43-related coupling in retinal vascular cells [81]. Early work using tracer coupling in retinal microvessels showed that diabetes reduces vascular cell coupling soon after diabetes induction, and insulin treatment prevents this reduction [82]. Cell and animal studies also show that high glucose reduces Connexin43 expression and gap junction intercellular communication in retinal microvascular endothelial cells and retinal pericytes [83,84]. In vivo, reduced Connexin43 expression promotes retinal vascular cell loss and increased permeability, and diabetes-associated Connexin43 downregulation associates with vascular lesions in both diabetic mice and diabetic human retinas [81,85].

The Connexin43 literature also indicates that effects can vary by cell type and by channel mode [86]. One study emphasizes loss of gap junction communication, while other studies highlight hemichannel opening and proinflammatory signaling under diabetic conditions [86]. One review summarizes this duality and discusses why interventions may need to target gap junction communication and hemichannel activity differently [86]. Together, impaired pericyte responsiveness and reduced endothelial–pericyte coupling can decrease the magnitude and the regional consistency of activity-evoked perfusion increases [69,70]. These changes can increase local perfusion variability during metabolic demand and can accelerate neurovascular unit instability in diabetic retinopathy [70]. In the amplifier model, sympathetic surges can further reduce this reserve and in-crease variability.

## 3. Diabetes-Driven NVU Dysfunction and NVC Impairment

### 3.1. Hyperglycemia, Oxidative Stress, and Energy Imbalance as Upstream Drivers

Diabetes exposes the retinal NVU to chronic metabolic stress [87]. Hyperglycemia increases mitochondrial reactive oxygen species and activates glucose-linked pathways (AGE–RAGE, polyol, hexosamine, and PKC) [87,88,89,90,91]. These pathways increase inflammatory signaling and weaken vascular homeostasis. This state can also increase the impact of sympathetic signaling. Endothelial injury and NO loss reduce buffering of adrenergic constriction, so the same catecholamine surge can cause a larger drop in vascular reserve [43,68].

**Figure 2:**
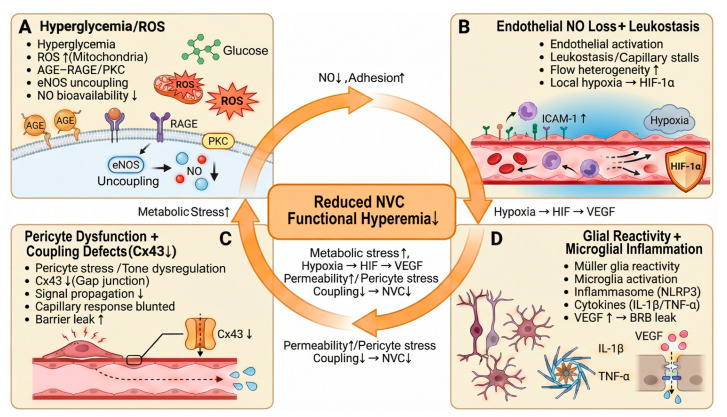
Diabetes-driven NVU dysfunction forms a self-reinforcing loop that reduces retinal neurovascular coupling. This schematic summarizes how multiple diabetes-related stresses converge on a weaker, less reliable activity-evoked blood-flow response in the retina. Diabetes reduces NO bioavailability, increases oxidative stress, and shifts glial and immune signaling, so functional hyperemia becomes blunted and more variable. (**A**) Hyperglycemia and oxidative stress: Hyperglycemia increases mitochondrial reactive oxygen species (ROS) and activates glucose-linked pathways, including AGE–RAGE and PKC signaling. ROS lowers effective NO by direct inactivation and by promoting endothelial dysfunction, including eNOS uncoupling. These changes reduce endothelial-dependent dilation and decrease vascular reserve. (**B**) Endothelial NO loss and leukostasis: Endothelial activation increases adhesion molecules such as ICAM-1, which promotes leukocyte adhesion and disrupts capillary transit. These events slow capillary flow and increase spatial variability in perfusion, which can impair stimulus-evoked blood-flow responses early in disease. Capillary stalls and uneven flow raise local hypoxia risk, which activates HIF programs and drives VEGF expression. (**C**) Pericyte dysfunction and coupling defects (Cx43↓): Diabetes reduces pericyte responsiveness and weakens coordination along the capillary wall. Connexin43 (Cx43)-dependent gap junction signaling supports signal spread across connected microvascular segments, and diabetes can impair this coupling. Reduced coupling weakens coordinated diameter control, increases perfusion variability, and increases permeability risk. (**D**) Glial reactivity and microglial inflammation: Müller glia become reactive early in diabetes and increase inflammatory and permeability-related outputs, including VEGF and cytokines. Microglia increase innate immune signaling and can engage inflammasome-related pathways, with higher IL-1β signaling and broader inflammatory effects on synapses and endothelium. These inflammatory signals further weaken barrier integrity and amplify endothelial activation. Central concept and feedback: Reduced functional hyperemia increases metabolic stress and hypoxia, and these stresses feedback to amplify oxidative stress, inflammation, VEGF signaling, and barrier dysfunction. This feedback loop accelerates NVU instability and further reduces NVC. Upward arrows indicate an increase, and downward arrows indicate a decrease.

Oxidative stress then reduces nitric oxide availability. Endothelial dysfunction can follow two common patterns [87]. First, reactive oxygen species react with nitric oxide and reduce its effective concentration [92]. Second, diabetes can increase arginase activity and limit L-arginine supply for nitric oxide synthase [92,93]. These changes reduce endothelial-dependent dilation and lower vascular reserve [93]. Endothelial activation also increases adhesion molecule expression and disrupts capillary transit [19,94,95]. Studies show early upregulation of ICAM-1 in diabetic retinal endothelium and an early rise in leukostasis [19,94,95]. These events slow capillary flow and increase spatial variability in perfusion [19,95]. This pattern can impair activity-evoked blood-flow responses even before obvious hemorrhage or microaneurysm formation (Figure 2).

### 3.2. Müller Glia as a Central Hub for Edema, Inflammation, and Coupling Defects

Endothelial dysfunction does not fully explain early functional changes [9,11]. Müller glia show early reactivity in diabetes and directly influence neurotransmitter clearance, ionic balance, and barrier signaling [96,97]. Diabetes can reduce glutamate uptake capacity in Müller cells, which increases extracellular glutamate exposure and raises neuronal stress. Diabetes can also disturb Müller cell water and potassium handling through changes in AQP4 and Kir4.1 localization. These changes promote edema and alter the extracellular microenvironment near vessels.

Reactive Müller glia also increase inflammatory and permeability-related outputs [97,98]. Multiple reviews and experimental studies report increases in VEGF and cytokines along with changes in iNOS and COX-2 signaling in diabetic retina [97,98,99]. These shifts weaken tight junction integrity and increase blood–retinal barrier vulnerability [100]. The vascular wall then becomes more sensitive to additional metabolic and inflammatory stress [98,100]. These Müller-driven changes matter for functional hyperemia [9,98]. Functional hyperemia needs rapid, time-locked signaling between neural activity and microvessels [9]. Diabetes can disrupt glial calcium handling and mediator balance, which reduces response amplitude and increases response delay [9,99]. Human imaging studies using capillary-resolved functional OCT angiography support early neurovascular dysfunction in diabetes [25].

### 3.3. Microglia and Innate Immune Activation: Synapse–Vessel Crosstalk

As diabetes progresses, innate immune signaling becomes more prominent [60]. Microglia respond to oxidative and metabolic danger signals and increase inflammatory output [60,101]. Several studies link diabetic retinal inflammation to inflammasome-related pathways, including NLRP3 activation and higher IL-1β signaling. This response can destabilize synaptic function and also increase endothelial activation [30,102,103,104,105].

Microglia can also affect microvascular stability through cell–cell signaling to mural cells [61,106]. Experimental work supports a mechanism in which microglial activation promotes pericyte injury and vascular dysfunction through inflammatory mediators and stress-response pathways [106,107]. This mechanism is consistent with the broader NVU model because pericyte loss and pericyte dysfunction reduce capillary-level control [107,108].

Recent work also links adrenergic signaling to microglial vascular responses in diabetes. Hyperglycemia can elevate circulating norepinephrine, and norepinephrine can reach the retina. β2-adrenergic receptor signaling in microglia can weaken microglial responses to microvascular injury and contribute to barrier breakdown. This finding provides a direct pathway that connects sympathetic biology to retinal NVU instability [46].

### 3.4. Neurovascular Crosstalk as an Integrated DR Mechanism

These pathways interact across cell types and create a self-reinforcing pattern [109,110]. Endothelial nitric oxide loss reduces vasodilatory reserve and increases adhesion signaling [26,111]. Capillary stalls and flow heterogeneity then increase local hypoxia risk [109]. Hypoxia-related signaling increases HIF-related programs and drives VEGF expression [112,113]. VEGF then increases permeability and further stresses the endothelium and pericytes [100,114].

Barrier disruption also increases exposure of retinal tissue to circulating factors [110]. This exposure can amplify microglial activation and sustain cytokine signaling [109]. Cytokines then intensify Müller glial reactivity and further destabilize neuronal and vascular function [110]. In this framework, reduced functional hyperemia is not only a marker [115]. Reduced functional hyperemia can increase metabolic stress and can accelerate the same inflammatory and vascular processes that drive progression [115].

This integrated view also clarifies why stress-related states may matter clinically. Sleep deprivation can impair endothelial function and can shift autonomic balance toward sympathetic dominance [38,116]. The retina expresses multiple adrenoceptor subtypes, and adrenergic signaling can become more consequential when endothelial function is already impaired [26,42]. Pain, anxiety, and perioperative stress often raise catecholamines, so these states may further reduce perfusion stability and increase inflammatory activity in the diabetic retina [117,118]. These points set the baseline for the amplifier model that we describe in Section 4.

## 4. Sympathetic Activation as an Amplifier of NVU Vulnerability in Diabetes

### 4.1. Adrenergic Control of Retinal Blood Flow and Vascular Tone

The retinal NVU links neuronal activity, glial signaling, and microvascular responses [10,13]. Neurons and Müller cells drive local signals that adjust arteriolar diameter, capillary resistance, and pericyte tone [10,13]. This process supports functional hyperemia and keeps tissue oxygenation stable during visual stimulation [10,119].

The retina has limited direct autonomic vasomotor innervation compared with many systemic vascular beds. Even so, the retinal vasculature expresses adrenergic receptors on vascular smooth muscle cells, endothelial cells, and pericytes [120]. Circulating catecholamines and local norepinephrine signaling can activate these receptors and shift baseline vascular tone [42,120,121]. In isolated mouse retinal arterioles (ex vivo), the endothelium buffers α1-mediated constriction under healthy conditions. Endothelial injury can unmask stronger α1-mediated constriction, which can lower retinal perfusion at the same perfusion pressure [43].

Adrenergic receptor subtypes appear to act through different routes in the retina. α1 signaling mainly controls vascular tone. In mouse retinal arterioles (ex vivo), α1-mediated constriction is buffered by the endothelium, and α1B becomes a key constrictor subtype after endothelial damage [43]. Retinal pericytes also contract in response to norepinephrine in vitro, which supports a capillary-level mechanism for catecholamine-driven tone shifts [122]. β2 signaling is supported more strongly in neuroimmune and barrier pathways. In a mouse study, hyperglycemia enhanced norepinephrine–ADRB2 signaling in microglia. This signaling weakened microglial responses to microvascular injury and increased barrier breakdown [46]. β2-adrenergic receptor-knockout mice also show a diabetic retinopathy phenotype [123]. Human studies show that sympathetic stimulation can change retinal vessel caliber, but these studies usually do not isolate α1 vs. β2 effects [121].

Human studies show that systemic sympathetic stimulation can change retinal vessel caliber [121]. Sustained isometric handgrip can induce measurable retinal vessel constriction in healthy adults [121]. Studies in diabetes report altered retinal vascular responses to sympathetic stimulation, and these responses relate to autonomic function tests in some cohorts [124].

Diabetes reduces vasodilator signaling and weakens neurovascular coupling [9]. Clinical studies using flicker stimulation show reduced retinal vasodilation in diabetes, and this impairment associates with retinopathy severity in some populations [18]. Reviews also describe reduced nitric oxide bioavailability, higher oxidative stress, and endothelial dysfunction as key contributors [9,26]. These changes reduce vascular reactivity and lower the capacity for activity-linked increases in blood flow [9]. This baseline state makes the retina more sensitive to additional sympathetic surges [9,121].

### 4.2. Pain, Anxiety, and Sleep Loss Converge on Sympathetic Physiology

Pain, anxiety, and sleep deprivation increase sympathetic outflow and raise catecholamine signaling [38,125,126]. Chronic pain conditions show sustained sympathetic involvement in many mechanistic models and clinical observations [127]. Anxiety states can also increase sympathetic nerve activity in human studies [125]. Sleep deprivation shifts autonomic balance toward sympathetic predominance and reduces vagal modulation in many datasets [128].

Sleep loss also impairs endothelial function and increases inflammatory signaling in humans [116,129]. Systematic reviews link sleep deprivation with worse endothelial-dependent vasodilation and higher vascular inflammation markers [116,129]. Immune studies also report that short sleep and sleep disruption are associated with a more proinflammatory baseline [129].

Diabetes changes the risk context for these stressors [130,131]. Diabetes increases endothelial dysfunction and reduces microvascular reactivity, so the same sympathetic surge can produce larger vasoconstriction and slower recovery [130,131,132]. Diabetes also increases the prevalence of autonomic neuropathy, including cardiovascular autonomic neuropathy, which disrupts autonomic control and limits physiological compensation during stress [133].

This combined physiology can destabilize retinal perfusion and NVU homeostasis [3,9]. Sympathetic surges can increase vasoconstrictor drive and can promote inflammatory signaling through adrenergic pathways in vascular and immune cells [134,135]. Diabetes-related NVU dysfunction already includes impaired neurovascular coupling, glial activation, and barrier vulnerability [3,9,11]. Reviews describe these processes as early features that can appear before advanced fundus lesions [3,11].

**Figure 3:**
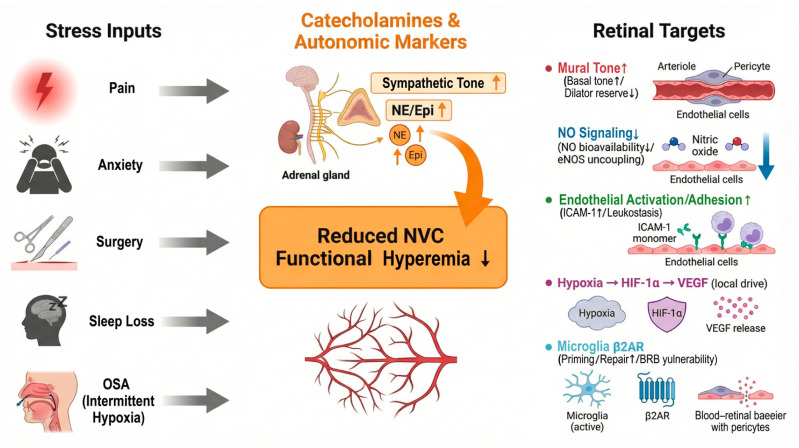
Stress- and sleep-related sympathetic activation converges on retinal flow control and reduces neurovascular coupling. This schematic shows how pain, anxiety, surgery, and sleep loss increase sympathetic outflow and circulating catecholamines and can amplify retinal NVU vulnerability in diabetes. Obstructive sleep apnea (OSA) adds intermittent hypoxia and further increases sympathetic tone, which can stress endothelial and immune pathways that support NVC. The middle panel highlights practical systemic readouts. Clinicians and researchers can quantify autonomic state with heart rate variability (HRV) at rest and during standardized challenges. Together, higher baseline tone, weaker NO signaling, endothelial activation and adhesion, and hypoxia-driven programs (HIF → VEGF) can reduce functional hyperemia. Arrows indicate the direction of influence, signaling, or downstream effects; Upward arrows indicate an increase, and downward arrows indicate a decrease.

Recent clinical work also supports early, subclinical microvascular change in diabetes using functional and imaging biomarkers [136,137]. Dynamic flicker-based testing can detect impaired hyperemic responses in early disease stages [137]. OCT angiography (OCTA) studies report lower vessel density and larger foveal avascular zone metrics with longer disease duration and higher HbA1c in type 1 diabetes populations [138,139]. A China-based hospital study also reports associations between higher cortisol levels and diabetic microvascular complications, including retinopathy, which supports a link between stress-axis activity and microvascular risk [140]. These findings support a model in which diabetes lowers vascular resilience, and stress-related sympathetic activation adds acute load on an already impaired NVU (Figure 3) [3,9,131].

Acute versus chronic stress exposures: Acute stress, such as a short surgical procedure, triggers a rapid sympathetic and hypothalamic–pituitary–adrenal response. Catecholamines and cortisol rise over minutes to hours, and these signals can change microvascular tone, inflammation, and metabolism during and soon after surgery [117,141]. Chronic stress, such as months of insomnia or persistent sleep loss, is different. It exposes the body to repeated nights of autonomic activation and low-grade inflammation. Studies in insomnia report altered cardiovascular autonomic activity, and sleep deprivation is linked with impaired endothelial function in humans [116,142]. In diabetes, both patterns may reduce retinal NVC. Acute stress is more likely to cause a transient NVC drop during a high-stress window. Chronic stress is more likely to shift the baseline state and reduce vascular reserve over time [116,141].

### 4.3. Mechanistic Routes Linking Sympathetic Drive to NVU and NVC Dysfunction

Most receptor-subtype-specific evidence comes from rodent in vivo studies, ex vivo arteriole preparations, or in vitro cell systems, while human data mainly show caliber or flow changes without isolating receptor subtype. Sympathetic activation can weaken retinal NVU and NVC because circulating catecholamines can act directly on retinal adrenergic receptors that are present in vascular and neural–glial compartments [42]. Diabetes can lower the safety margin of this system because many studies show that retinal vascular reactivity and stimulus-evoked hyperemia are already reduced in diabetes, even in early stages [9,18,71]. This background makes sympathetic surges more likely to push retinal perfusion control into a state where blood delivery no longer matches neural demand [9].

Adrenergic tone can reduce vasodilatory reserve by raising baseline resistance in upstream arterioles and in the microvascular network [9,43]. Experimental work in mouse retinal arterioles shows that α1-adrenergic vasoconstriction is normally buffered by the endothelium and that endothelial injury strengthens this constrictor response [43]. Human autonomic stimulation can also constrict retinal vessels during isometric handgrip, which supports a link between systemic sympathetic activation and retinal vessel caliber [143]. When baseline tone rises, the same neural stimulus produces a smaller diameter change and a smaller flow increase [9]. Clinical studies using flicker stimulation report reduced dilation responses in diabetes, which is consistent with limited reserve and impaired coupling [9,18].

Sympathetic drive can also worsen endothelial NO signaling, which is important for basal retinal vascular tone and for flicker-induced dilation in humans [144]. Oxidative stress lowers NO bioavailability because reactive oxygen species inactivate NO and can promote eNOS uncoupling, which further increases oxidative load and weakens endothelial function [145]. Diabetes already shifts the retina toward higher oxidative stress and lower NO effectiveness, so additional stress-related vascular signaling can further reduce NO-dependent dilation and can strengthen endothelial activation [9,26]. Reduced NO signaling also removes a brake on leukocyte–endothelial interactions, which increases the chance of capillary flow slowing during stress [146].

Barrier stress and leukocyte adhesion provide another route that links sympathetic-associated stress physiology to NVC failure [9,94]. In experimental diabetes, ICAM-1 blockade reduces retinal leukostasis and strongly reduces blood–retinal barrier breakdown, which supports a causal role for leukocyte adhesion in early vascular leakage [94]. VEGF can induce ICAM-1 and promote early diabetic retinal leukocyte adhesion, which connects hypoxia-responsive signaling to inflammatory capillary plugging [147]. The AGE–RAGE axis can also drive leukostasis and barrier injury in the diabetic retina, which adds a metabolic amplifier to this inflammatory route [148]. When leukocytes adhere, and capillary transit becomes uneven, local oxygen delivery becomes less uniform, and that mismatch can further increase VEGF, oxidative stress, and cytokine output [9,26,147]. This feedback can further reduce the precision of stimulus-evoked hyperemia [9].

Catecholamines and glucocorticoids at the blood–retinal barrier: Diabetic retinopathy has a chronic inflammatory component, and this inflammation contributes to BRB breakdown [149,150]. Catecholamines can act on adrenergic receptors in retinal barrier cells. In a high-glucose RPE model, α1D-adrenergic receptor blockade prevented epithelial barrier damage, which supports a direct link between adrenergic signaling and barrier vulnerability under diabetic-like stress [151]. In retinal endothelial and Müller cells, β-adrenoceptor signaling can support hypoxia-driven VEGF upregulation, which can favor leakage when the retina is stressed. Glucocorticoids often push in the opposite direction [152,153]. In primary retinal endothelial cells, glucocorticoids increased occludin and claudin-5 and improved endothelial barrier properties. Glucocorticoids can also limit endothelial inflammatory activation through glucocorticoid receptor-dependent control of NF-κB-related signaling. Finally, adrenergic and glucocorticoid pathways can influence each other at the receptor level. Studies in other tissues show that β2-adrenergic signals can modulate glucocorticoid receptor function, and glucocorticoids can increase β2-adrenergic receptor expression and reduce receptor desensitization [154].

Glial and immune state changes can connect sympathetic signals to NVC through NVU regulation of capillary tone and vasoactive mediator release [9]. Microglia can regulate retinal capillary diameter via fractalkine signaling, and early diabetes alters this control by increasing angiotensinogen and by engaging renin–angiotensin signaling; candesartan can abolish the microglia-driven vasoconstriction in this model [155]. Reviews of early diabetic NVC anomalies emphasize that altered glial–vascular signaling is a plausible driver of reduced functional hyperemia before overt retinopathy [9]. Adrenergic signaling can also intersect with Müller glia–vascular biology, because β2-adrenergic receptor deletion in mice produces retinal changes that resemble diabetic phenotypes and alters insulin-related signaling pathways in the retina [9,123]. These findings support a model in which sympathetic surges add a stress input to a retina that already has higher inflammatory readiness, altered glial signaling, and reduced vascular flexibility [9,26].

This integrated view also clarifies what can be measured in people and what can be tested in models. Dynamic OCTA studies show impaired retinal vascular reactivity in type 1 diabetes without clinical retinopathy, which provides a human readout of early functional impairment [71]. Large community-based OCTA data from China also show that diabetes and higher fasting glucose correlate with reduced macular perfusion and vascular density, which supports the idea that microvascular vulnerability can be present before classic fundus signs [156,157]. Autonomic imbalance is common in diabetes and is recognized as a microvascular complication, so autonomic measures can serve as a systemic context when interpreting retinal NVC outcomes [133,158]. Together, these studies motivate endpoints that link sympathetic drive to retinal NVU/NVC risk: baseline vessel caliber and tone, flicker- or task-evoked dilation, NO-related endothelial function, adhesion and barrier markers, and indices of glial inflammatory state aligned to an NVC readout [9].

## 5. Autonomic Biomarkers and Human Evidence

Human studies can measure autonomic function and retinal neurovascular coupling in the same person [72]. Autonomic function can be assessed with heart rate variability at rest and during standardized challenges such as active standing or tilt testing [133]. These protocols probe baroreflex control and help identify diabetic cardiovascular autonomic neuropathy [133]. Retinal NVC can be assessed with flicker stimulation while measuring vessel diameter and flow [9,159]. Researchers most often use dynamic retinal vessel analysis for flicker-induced vasodilation, and they also use Doppler OCT or related OCT-based approaches for blood-flow and velocity changes [72,159,160]. Standard operating procedures for dynamic vessel analysis have improved comparability across cohorts [160].

Clinical-trial applicability and protocol standardization: Flicker-evoked retinal neurovascular coupling can be tested in humans with DVA and related approaches [161]. In the common DVA protocol, the test uses three identical cycles (50 s baseline, 20 s flicker at 12.5 Hz, and 80 s recovery) after pupil dilation, and it measures flicker-light-induced dilation in an arteriole and a venule as a non-invasive functional readout [161]. DVA measurements show high reproducibility in re-analysis, and prior work has also reported good day-to-day reproducibility [160]. Many human clinical studies have used flicker-induced retinal vasodilation measured by DVA in diabetes and diabetic retinopathy, which supports its feasibility in clinical research settings [16,21]. Other platforms can quantify the same stimulus response as blood-flow changes, such as Doppler OCT measurements of flicker-induced increases in total retinal blood flow [159]. However, methods differ across diameter-based DVA, Doppler OCT flow metrics, and capillary-level OCTA, and OCTA reviews emphasize the need to harmonize acquisition, analysis, metrics, and reporting to make results comparable across studies and devices [162,163]. For multicenter designs, trials should use site training/certification and centralized quality control or reading to reduce variability and to handle image artifacts consistently [160,164].

Work from multiple groups shows that flicker responses are reduced in diabetes and tend to worsen as retinopathy advances [9,18]. A clinical review that synthesized human and animal evidence concluded that impaired retinal functional hyperemia can appear early and may occur before clear fundus lesions in some patients [9]. Consistent with this view, studies in patients with diabetes without clinical DR reported reduced flicker-induced dilation compared with controls [18,165]. These results support the idea that retinal NVC testing can detect functional impairment when standard structural signs are still limited [9,165].

Prospective data also support clinical relevance. In a longitudinal study, a reduced flicker-induced vasodilation response predicted later DR progression, which links an early functional deficit to later disease worsening [22]. Other human work has extended the readouts beyond diameter and flow. For example, retinal oxygen metrics measured during flicker show DR-stage-dependent differences in oxygen saturation and oxygen extraction fraction, which adds metabolic context to perfusion readouts [166].

The causality between sympathetic overactivity and NVU damage in diabetes is unresolved. Prospective human data link a reduced flicker-induced dilation response to incident DR and to DR progression over follow-up [22]. Reviews also describe impaired retinal functional hyperemia as an early feature in diabetes, and some patients show impairment before clear fundus lesions [9]. These links support clinical relevance, but they do not define cause. In the experimental and clinical literature, the direction between vascular dysfunction and structural change is still not settled. One study notes that it is unclear whether altered vascular structure leads to flow dysregulation in diabetes, or the reverse, and it highlights limits of cross-sectional human data [167]. In a longitudinal type 2 diabetes mouse study, flow responses and neurovascular coupling deficits appeared before clear neural dysfunction on an ERG [167]. Clinical work also states that the reason for the diminished flicker hyperemic response is not fully explained, and it discusses both endothelial and neural hypotheses [72]. We therefore frame sympathetic overactivity in two ways. It may act as a primary driver in some settings. It may also act as an amplifier when diabetes has already reduced vascular reserve. Future studies should test time order in longitudinal cohorts. These studies should pair NVC testing with autonomic measures and OCT/OCTA endpoints. Early detection and early treatment of abnormal retinal circulation may help limit later DR development [167].

Direct human evidence that combines autonomic testing with retinal NVC is still limited, but it is emerging [72]. Hommer and colleagues measured HRV during an orthostatic challenge and measured flicker-evoked retinal hyperemia using Doppler OCT in the same visit [72]. They reported a DR-stage-dependent reduction in flicker hyperemia and an altered autonomic response profile in diabetes. They also reported that cross-sectional HRV indices did not show a strict one-to-one mapping to the size of the flicker blood-flow response. These results fit a model in which autonomic dysfunction and retinal NVC impairment can progress in parallel, while local retinal factors still shape the final magnitude of the NVC response [72].

Current mechanistic data support several ways that systemic autonomic state can influence the retinal NVU. The retina expresses adrenergic receptor subtypes across vascular and glial compartments, so circulating catecholamines can act directly on retinal cells [42]. Sympathetic activation can increase baseline microvascular tone through adrenergic signaling in mural cells, and this change can reduce the capacity and speed of flicker-evoked dilation [42]. Diabetes can also reduce nitric oxide bioavailability through oxidative stress and endothelial dysfunction, so the same sympathetic surge can have a larger functional impact when vascular reserve is already reduced [130,144]. Recent experimental work also provides a direct neuroimmune link. In a diabetic context, norepinephrine signaling through microglial β2-adrenergic receptors was reported to impair microglial responses to microvascular injury and to promote blood–retinal barrier disruption, which can destabilize perfusion control and inflammatory balance [46].

**Figure 4:**
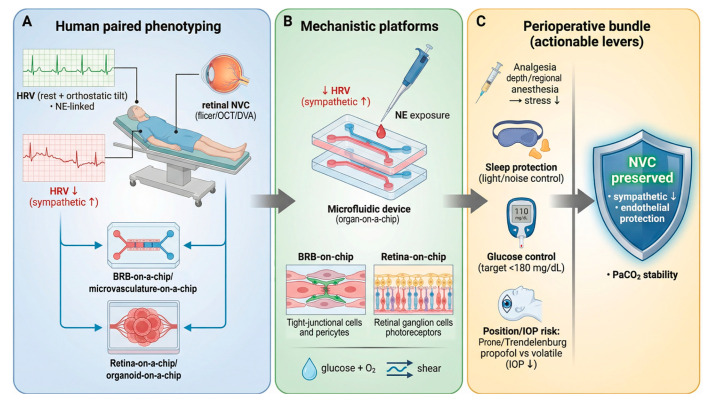
Translational measurement toolbox linking autonomic state, retinal neurovascular coupling, and perioperative levers to preserve NVC in diabetes. (**A**) Human paired phenotyping: This panel pairs systemic autonomic assessment with a retinal functional readout. The protocol measures heart rate variability (HRV) at rest and during an orthostatic challenge (tilt/standing). Lower HRV can reflect reduced autonomic flexibility and is often used as a marker of relative sympathetic predominance. It is not a direct measure of catecholamine exposure. The same visit can quantify retinal neurovascular coupling (NVC) with flicker stimulation using dynamic vessel analysis (DVA) and/or OCT-based flow methods. These paired measures assess systemic autonomic state alongside retinal stimulus-evoked hyperemia in the same person. This design can test whether the two signals vary together. (**B**) Mechanistic platforms: This panel translates clinical pairing into controllable microphysiological systems. Microfluidic organ-on-a-chip models can define NE exposure as an input while controlling key context variables (e.g., glucose, oxygenation, and shear stress). Barrier-focused blood–retinal barrier (BRB)-on-chip designs model tight-junction-forming vascular cells and pericytes, while retina-on-a-chip models include neuronal components. These platforms support time-resolved readouts that map how catecholamine input propagates across vascular, barrier, and neural–glial compartments. (**C**) Perioperative bundle (actionable levers): This panel summarizes practical perioperative targets that can reduce stress signaling and protect retinal perfusion control. Clinicians can blunt sympathetic surges with adequate analgesia and appropriate anesthetic depth or regional techniques. Clinicians can protect sleep (light/noise control), maintain perioperative glucose near guideline targets (often <180 mg/dL), and reduce position-related intraocular pressure (IOP) risk in prone/Trendelenburg cases (including consideration of propofol vs. volatile techniques when appropriate). Clinicians can also avoid large PaCO_2_ swings because CO_2_ shifts retinal vascular tone. Together, these steps aim to preserve NVC by lowering sympathetic load and supporting endothelial function. Arrows indicate the direction of association, experimental flow, or proposed intervention effects; Upward arrows indicate an increase, and downward arrows indicate a decrease.

These points suggest practical translational strategies. A useful human design can pair (1) resting HRV and challenge-based HRV, (2) flicker-based NVC testing with DVA or Doppler OCT, and (3) structural OCT/OCTA markers of vascular dropout and neuroretinal thinning. This combined approach can separate “systemic autonomic inflexibility” from “local retinal vascular unresponsiveness,” even when the two do not correlate tightly in cross-section [72]. Functional OCT angiography with capillary-level resolution is also becoming a direct tool to test early neurovascular dysfunction in diabetes [71]. Finally, sleep loss is a relevant and measurable modifier in perioperative and pain settings. Human laboratory data show that sleep deprivation can alter HRV and impair endothelial function, which supports its role as a plausible upstream stressor in patients who already have limited vascular reserve (Figure 4) [116,128].

Flicker ERGs as a neural companion to NVC testing: The ERG provides an objective measure of retinal electrical responses, and it can be used to assess neural integrity during visual stimulation [168]. The ISCEV Standard includes a light-adapted ERG recorded at a frequency close to 30 Hz, which supports consistent acquisition and reporting across studies [169]. The 30 Hz flicker ERG is mainly generated by ON and OFF bipolar cells in the cone pathway [168]. In diabetes, a study report delayed 30 Hz flicker implicit time, and the delay increased with retinopathy severity [170]. A clinical review also reports that flicker ERG amplitude reduction and implicit-time delay are small in mild- or no-DR conditions, but they are larger in moderate to severe DR [168]. Diabetes also affects inner retinal circuits. Oscillatory potentials are reduced in diabetic eyes, including eyes without clinically detectable DR, and this pattern suggests early amacrine cell involvement [171]. In translational protocols, trials can record flicker ERG (and/or oscillatory potentials) together with flicker-evoked NVC. This pairing can help interpret a small NVC response. If the ERG is abnormal while NVC is small, the data support reduced neural drive. If the ERG is preserved while NVC is small, the data support a vascular execution deficit. This logic is consistent with human evidence showing reduced flicker vascular responses in early type 1 diabetes despite preserved pattern ERG [165].

## 6. Perioperative and Anesthesiology-Relevant Context

### 6.1. Perioperative Stress Response and Microcirculatory Consequences

Surgery activates sympathetic outflow and the hypothalamic–pituitary–adrenal axis [117,141]. This perioperative stress response is an acute exposure, which differs from chronic stressors such as prolonged insomnia. Tissue injury and pain increase sympathetic efferent activity and raise circulating catecholamines [141]. Hypothalamic signaling also increases ACTH and raises cortisol [117,141]. These responses also change glucose handling and immune signaling, so many patients develop perioperative hyperglycemia and a proinflammatory state [117,172,173].

These neuroendocrine and inflammatory changes can alter microcirculation even when systemic blood pressure looks acceptable [174,175]. Perioperative microcirculatory dysfunction can involve reduced capillary density, impaired flow distribution, and slower capillary transit [174,176,177]. Reviews in perioperative medicine emphasize that microcirculatory changes can contribute to organ stress and can remain “hidden” if clinicians only track macrocirculatory variables [174,175].

Catecholamines can shift baseline mural cell tone toward constriction [141,178]. α1-adrenergic signaling increases intracellular calcium in vascular smooth muscle and increases contractile tone [178]. The retina also expresses adrenergic receptor subtypes, so systemic catecholamines can act on retinal vascular and neural–glial elements [42]. In the retinal capillary bed, pericytes are central for local diameter control and flow distribution [115,179]. An experimental study in the diabetic retina showed reduced pericyte responsiveness and impaired propagation of vasomotor responses, and further associated the propagation deficit with reduced Connexin43-mediated gap junction coupling [70]. These findings support a simple perioperative concern: higher sympathetic tone can raise baseline vascular tone, and diabetes can reduce the ability of the capillary network to redistribute flow when demand changes [70,174].

Endothelial nitric oxide signaling is another limiting factor for microvascular dilation [9]. Diabetes reduces nitric oxide availability through oxidative stress and related endothelial dysfunction [9,180]. Perioperative stress can add oxidative and inflammatory pressure to the vessel wall, which can further reduce effective nitric oxide signaling [117,174,180]. Perioperative microvascular injury also involves the endothelial glycocalyx, which supports barrier function and reduces leukocyte adhesion [181]. Reviews and perioperative studies describe glycocalyx shedding during major surgery, especially in settings with inflammation and ischemia–reperfusion [182,183]. The retina has its own glycocalyx-relevant biology, and recent work links glycocalyx loss to endothelial dysfunction in retinal disease settings, including diabetic retinopathy [184,185].

Inflammation-driven leukocyte–endothelial interactions can directly disturb capillary flow [174,181]. Surgical stress can increase endothelial activation and can promote leukocyte adhesion [117,174]. Glycocalyx degradation can also increase leukocyte and platelet adhesion and can increase edema risk [181,182]. Diabetes already increases susceptibility to leukostasis in the retinal circulation [94]. Classic experimental work showed that ICAM-1 blockade prevents diabetic retinal leukostasis and reduces vascular leakage, which supports a causal role for leukocyte adhesion in early microvascular dysfunction [94]. This mechanism fits perioperative risk logic because any added perioperative endothelial activation can increase the chance of capillary plugging in a vascular bed that already has reduced reserve [94,174,175].

Perioperative hyperglycemia and coagulation shifts can also worsen microvascular flow quality [172,173,186]. Hyperglycemia in type 2 diabetes is associated with higher blood viscosity in human studies [187,188]. Diabetes is also associated with reduced red blood cell deformability, which can increase microvascular resistance and impair oxygen delivery [189,190]. These hemorheological changes can increase capillary transit time heterogeneity, so tissue oxygen delivery can fall even without a large change in systemic pressure [191,192,193].

These mechanisms make perioperative management relevant for retinal microcirculation, especially in patients with diabetes [117,186]. Evidence from anesthesia research shows that regional anesthesia can attenuate the neuroendocrine stress response and reduce cortisol and catecholamine release [194]. Multimodal analgesia can also reduce stress responses by reducing nociceptive input and sympathetic activation [117]. These approaches may help stabilize microvascular tone and inflammatory activation, but retinal-specific outcome studies are still needed.

### 6.2. Anesthesia Modality, Ocular Perfusion Surrogates, and Why These Matter for the Diabetic Retina

Direct perioperative measurements of retinal NVC remain rare, but perioperative physiology changes several determinants of ocular blood flow. Ocular perfusion pressure is often estimated as mean arterial pressure minus intraocular pressure (IOP) [195]. Many anesthetic and surgical factors change mean arterial pressure, IOP, or both [194]. These shifts can reduce the driving pressure for retinal and optic nerve head perfusion, even when systemic blood pressure appears acceptable [195,196].

Patient position has a consistent effect on IOP [196,197,198]. Prone positioning increases IOP during anesthesia, so ocular perfusion pressure can fall during long prone cases [196,199]. Steep Trendelenburg and pneumoperitoneum also increase IOP, and the IOP rise can be clinically meaningful when exposure is prolonged [195,197,200]. Lateral decubitus positioning can increase IOP in the dependent eye, and the increase can depend on the anesthetic regimen [198]. These findings support the use of IOP and ocular perfusion pressure as practical perioperative surrogates when direct retinal flow measures are not available [195].

Anesthetic modality can modify these pressure effects [198,200]. Randomized trials during steep Trendelenburg show that propofol-based anesthesia can blunt the IOP rise compared with sevoflurane [199]. A controlled study in lateral decubitus positioning found that propofol did not show the same IOP increase that occurred with sevoflurane at one hour after the position change [198]. These differences matter most for patients who start with limited microvascular reserve, because a smaller IOP increase can preserve ocular perfusion pressure during the case [11].

Ventilation and arterial carbon dioxide also affect retinal vascular tone [201]. A human study showed that inhaled CO_2_ causes vasodilation in retinal arteries and veins and increases retinal blood flow [201]. Perioperative ventilation can shift PaCO_2_, so ventilator targets can change retinal vascular reactivity during anesthesia [201]. In contrast, some ventilator settings mainly influence venous return and intrathoracic pressure, which can indirectly affect IOP through venous congestion [202]. Trials in Trendelenburg suggest that low levels of PEEP do not necessarily increase IOP in that setting, but the net effect still depends on position, pneumoperitoneum, and hemodynamics [195,202]. OSA can add a mechanical effect beyond intermittent hypoxia. During obstructed breathing, inspiratory effort creates large negative intrathoracic pressure swings. These pleural pressure swings change venous return and systemic hemodynamics, so they can also shift ocular venous dynamics and IOP. A Mueller-maneuver model of obstructed inspiration showed a dose-dependent acute IOP decrease with stronger negative pressure [203]. Continuous IOP monitoring during sleep also shows that severe OSAS has longer periods of nocturnal IOP elevation than mild/moderate disease [204]. Reviews further report higher IOP in some OSA cohorts and propose increased episcleral venous pressure as one contributor, especially in patients with higher BMI [205].

These perioperative variables matter more in diabetes because diabetes weakens the buffering capacity of the retinal neurovascular unit [11]. Reviews of retinal NVC in diabetes report reduced flicker-induced vasodilation in both type 1 and type 2 diabetes, including cases with no or mild clinical retinopathy [11]. It also highlights early anomalies in retinal vasoregulation during diabetes and links these anomalies to impaired activity-evoked perfusion responses [11]. Large community-based OCTA data from China show that diabetes and higher fasting glucose are associated with lower macular perfusion and vascular density, which supports reduced structural reserve in the diabetic retinal microcirculation [156].

A mechanistic chain can connect perioperative physiology to NVC impairment in a way that matches these constraints. Surgical pain and stress increase sympathetic output, and sympathetic activation increases circulating catecholamines during the perioperative period [117]. Catecholamines can raise constrictor tone, which can reduce microvascular perfusion uniformity when ocular perfusion pressure is already reduced by IOP elevation or reduced mean arterial pressure [117,195]. Perioperative hyperglycemia can further stress the endothelium. Acute hyperglycemia can damage the endothelial glycocalyx and coincide with endothelial dysfunction and coagulation activation in humans [206]. Perioperative guidance documents emphasize avoiding marked hyperglycemia, and widely used recommendations target intraoperative glucose below 180 mg/dL for many patients [207,208]. This combined context can reduce nitric-oxide-mediated relaxation and can promote an inflammatory endothelial state, which can further weaken stimulus-evoked dilation in a diabetic retina [206].

This framework supports specific perioperative levers that clinicians already use. Clinicians can reduce nociceptive input and blunt sympathetic surges with adequate analgesia and stable anesthetic depth [117]. Clinicians can limit IOP elevation by minimizing prolonged steep Trendelenburg or prolonged prone exposure when feasible, and by managing venous congestion and excessive fluid loading during high-risk cases [195]. Clinicians can consider anesthetic techniques that attenuate IOP increases in positions that raise IOP, based on trial data comparing propofol and volatile anesthesia [198,200]. Clinicians can also avoid large PaCO_2_ swings because CO_2_ changes retinal blood flow and vascular tone in humans [201]. Clinicians can manage perioperative glucose using guideline-based targets, including Chinese perioperative glucose guidelines, because hyperglycemia can rapidly impair endothelial function and can increase vascular risk [206,207,208].

## 7. Experimental Platforms to Test the Sympathetic–NVU Hypothesis

### 7.1. Retinal Organ-on-a-Chip and Related Microphysiological Systems

Animal models remain essential in diabetic retinopathy research [209]. However, animal models often combine several systemic changes at the same time [209]. Pain, sleep loss, perioperative stress, and anxiety can change catecholamines, cortisol, blood pressure, CO_2_, and inflammatory tone together. These combined changes can make causal interpretation difficult. Microphysiological systems can address this limitation because these systems allow controlled changes in glucose, oxygen tension, perfusion pressure, shear stress, and immune exposure while preserving key NVU-related cell contacts [210]. Studies in retina-on-a-chip and barrier-on-chip platforms support this approach and provide human-cell-based readouts that are relevant to translational testing [211,212].

Inner blood–retinal barrier and retinal microvasculature chips provide a practical entry point for stress-related questions that focus on perfusion stability, barrier function, and leukocyte–endothelial interactions [211,212]. Ragelle and colleagues established a human retinal microvasculature-on-a-chip with integrated flow using human retinal microvascular endothelial cells, and the model supports quantitative permeability readouts under defined perfusion conditions [213]. More recent work developed a diabetic inner blood–retinal barrier-on-a-chip that reproduces early disease-like phenotypes, including pericyte loss, vascular regression, and inflammatory factor production under diabetic stimulation [214]. These systems allow direct tests of adrenergic inputs under controlled baseline states. A study design can set a diabetic baseline first and then add norepinephrine or epinephrine with or without inflammatory cues. The same device can quantify microvessel diameter, flow distribution, permeability to tracers, junctional protein organization, and leukocyte adhesion under flow. The design can also include pharmacologic controls such as α- or β-adrenergic antagonists to test receptor specificity.

Outer blood–retinal barrier chips provide complementary information because the outer barrier depends on RPE structure and its interface with a vascular compartment. Arık and colleagues reported an outer blood–retinal barrier organ-on-a-chip with an RPE layer and an endothelial microvessel separated by a semipermeable membrane and validated barrier and vascular structure readouts [215]. Outer-barrier platforms can test whether catecholamines and glucocorticoids alter RPE barrier properties or change endothelial activation under diabetic-like stress. These platforms can also support transport and permeability studies that are relevant to drug exposure and barrier vulnerability.

Retina-on-a-chip platforms with neural components address a different part of the mechanism because these platforms allow direct measurement of neural and glial responses that precede vascular changes in neurovascular coupling. Achberger and colleagues merged retinal organoid tissue with organ-on-a-chip design and created a human retina-on-a-chip platform that integrates multiple retinal cell types and supports layered tissue organization [216]. A later study used a stem-cell-based retina-on-a-chip platform to support translational testing in the context of retinal gene therapy vectors, which further supports the feasibility and robustness of these systems for controlled perturbation studies [217]. For the sympathetic–NVU question, these platforms can test whether catecholamines change Müller glia calcium dynamics, gliotransmitter release, and metabolic outputs that shape vascular signaling. A platform that includes a vascular compartment can then extend the readout to activity-linked flow changes under controlled glucose and oxygen conditions.

Microfluidic intact-retina and slice-based devices remain useful when a study needs fast, localized perturbation and clear time ordering. Dodson and colleagues reported a retina-on-a-chip platform for whole retina or tissue slices with point-access reagent delivery which supports spatially targeted stimulation while maintaining culture stability over multiple days [218]. This approach can apply norepinephrine to defined regions while holding glucose and oxygen constant. This approach can separate immediate vascular wall and mural cell responses from slower glial and immune responses across hours. Reviews of microfluidic methods for intact tissues support this type of design for mechanistic dissection in complex neural tissues [219]. These platforms do not replace in vivo models, and each platform has limits in maturity, innervation, and systemic physiology. However, these platforms allow controlled exposure to catecholamines and glucocorticoids under defined diabetic baselines. This control is useful for testing whether sympathetic mediators reduce microvascular reserve, increase flow heterogeneity, increase endothelial activation, and shift glial and immune set points. Studies that combine these human-cell-based platforms with targeted in vivo validation can provide stronger causal evidence for stress-related amplification of retinal NVU vulnerability in diabetes.

### 7.2. Targets and Readouts That Map “Who Affects Whom”

These mechanistic sections set a simple requirement for experimental platforms. A platform should treat catecholamines as the defined input and then measure time-ordered outputs across the neurovascular unit. Retinal adrenergic signaling is plausible because the retina expresses α- and β-adrenergic receptors across vascular and neural–glial compartments [42].

The first output should sit at the vascular tone node. Retinal arterioles can constrict through α1-adrenergic signaling, and endothelial impairment can unmask stronger adrenergic constriction [43]. Böhmer and colleagues showed that α1-mediated constriction becomes prominent when the endothelium is damaged, and they identified α1B as a key subtype in mouse retinal arterioles [43]. Retinal pericytes can also contract in response to norepinephrine in vitro, which supports a capillary-level route for catecholamine-driven tone shifts [122]. In microphysiological systems, the core readouts should include vessel diameter tracking, pericyte calcium imaging, and flow mapping across capillary networks. A platform should also include perturbations that test direction and specificity. An α1 antagonist can test the constrictor arm, and downstream contractility tests can target Rho-family signaling that regulates pericyte contraction [220,221].

The next output should sit at the endothelial signaling node. Endothelial nitric oxide signaling supports rapid dilation and helps keep the endothelial surface less adhesive [144,222]. Diabetes often weakens this axis, so small additional stressors can reduce dilation capacity further [11]. In platforms, the endothelial panel should include nitric oxide reporters, eNOS phosphorylation state, and oxidative stress readouts that directly relate to nitric oxide loss. A platform should then link these endothelial changes to functional dilation capacity under a defined activity trigger. Human evidence supports this structure because retinal flicker responses are reduced with diabetic retinopathy severity, and lower flicker-induced dilation is linked to a higher short-term risk of retinopathy progression [19,22].

The third output should sit at the barrier and leukocyte–endothelium interaction node. Endothelial activation can increase adhesion programs such as ICAM-1 and VCAM-1, which can raise leukocyte adhesion and slow capillary transit [95]. This step creates flow stalls and higher flow heterogeneity [95]. Those changes can create local hypoxia and local inflammatory signaling, which then worsen barrier integrity. Experimental work in diabetic retinopathy supports leukostasis and adhesion as early contributors that can be modified by blocking adhesion pathways [94,95]. In platforms, you can measure barrier leaks with tracer permeability and tight junction localization, and you can quantify leukocyte adhesion and capillary stalls under controlled shear. If the platform supports electrodes, TEER can add a continuous barrier readout and can improve throughput for intervention screening.

The fourth output should sit at the glial and immune state node, because glia and microglia can shape vascular responses. Microglia can regulate retinal vascular tone, and early diabetes can change this microglial vasoregulation [11,155]. Catecholamines can also act on immune cells through adrenergic receptors and can change cytokine programs [223]. In the retina, Müller glia sit at the blood–retinal barrier interface and can drive or modulate inflammatory signaling during stress [97]. So, a platform should measure microglial activation markers, cytokine outputs, and Müller glia calcium dynamics or mediator release. A platform should then test whether these glial changes occur before, and then predict, the vascular coupling change. That time ordering is what turns correlation into a causal map.

A final requirement is that the system should measure neurovascular coupling rather than general vascular injury. The platform needs an activity trigger and a matched flow or diameter readout. Flicker stimulation is a clinically used trigger, and it produces measurable retinal vessel dilation in humans [19,160]. A study using dynamic vessel analysis showed reduced flicker-induced dilation in diabetes and further reduction with more severe diabetic retinopathy [19]. In model systems, patterned light stimulation, optogenetic activation, or controlled neuronal activation can serve the same role, as long as the platform records vessel diameter and flow with sufficient time resolution. Recent platform work supports the feasibility of this staged design. Reviews of retina-on-a-chip systems describe designs that include perfused vascular channels, retinal barrier features, and multicell cocultures that better match retinal microenvironments than simple monocultures [212]. This progress matters because it allows experiments that combine catecholamine exposure, diabetic context, and sequential readouts in one system. It also allows alignment with human endpoints. Large cohort imaging from China shows that OCTA detects retinal microvascular alterations associated with diabetes and prediabetes, which supports the idea that measurable microvascular vulnerability can appear before advanced fundus findings [156,224].

## 8. Therapeutic and Preventive Opportunities

### 8.1. Classical DR Care, Plus an NVU Target That Sympathetic Tone Can Shift

Current prevention and treatment of diabetic retinopathy still rely on measures with strong clinical evidence. Clinicians reduce risk by improving glycemic control and by treating blood pressure and lipid risk. Intensive glycemic control reduces retinopathy development and slows progression in long-term follow-up cohorts [225,226,227]. Blood pressure control also reduces retinopathy progression in type 2 diabetes [228]. Fenofibrate reduces retinopathy progression signals and lowers the need for laser therapy in large, randomized trials [229,230]. When macular edema or proliferative features dominate, intravitreal anti-VEGF therapy provides strong functional benefit and remains central in ophthalmic care [231,232].

These established approaches mainly target metabolic drivers and late vascular outcomes. The NVU framework adds an additional therapeutic goal. Clinicians and researchers can also aim to maintain coordinated neural–glial–vascular function. Retinal functional hyperemia is a practical readout because it reflects endothelial responsiveness, pericyte behavior, and glial signaling within the same test [5,9]. Large mechanistic reviews support the view that early NVU dysfunction can appear before obvious hemorrhage and can contribute to later vascular injury [3,5].

Sympathetic tone becomes relevant because it can modify several NVU-sensitive processes. Retinal vessels and retinal cells express adrenergic receptors, which create direct entry points for catecholamine signaling [42]. Catecholamines can increase mural cell calcium signaling and raise baseline vascular tone [42,43]. This change can reduce activity-evoked dilation when endothelial nitric oxide signaling is already weakened by diabetes-related oxidative stress [9,26]. Sympathetic activation can also interact with inflammatory pathways. Recent work links β2-adrenergic signaling in retinal microglia to impaired vascular repair responses and barrier vulnerability in diabetic settings [46].

This logic supports an additive strategy rather than a replacement strategy. Standard DR care remains necessary. At the same time, studies can test whether reducing sustained sympathetic activation improves functional hyperemia and stabilizes NVU behavior under diabetic conditions. This approach fits clinical states that often raise sympathetic tone, including pain, anxiety, and sleep loss [125,128,233].

Not all studies support β-adrenergic blockade as retinal protection in diabetes. In diabetic Ren-2 rats, valsartan reduced retinal vascular pathology, but atenolol had no effect [234]. In the UKPDS, retinopathy progression was similar in patients treated mainly with atenolol or captopril, and the authors reported no drug-specific benefit [235]. In a pilot study of proliferative diabetic retinopathy, oral propranolol for 12 weeks did not reduce retinal neovascularization [236]. An earlier physiology study in diabetic subjects reported that propranolol did not change retinal vessel diameter or volumetric flow [237]. These differences likely reflect variation in receptor subtype targeting, exposure, disease stage, and study endpoints.

### 8.2. Perioperative Care as a Real-World Lever for Stress, Microcirculation, and NVU Stability

The perioperative period creates a predictable rise in sympathetic output and stress hormones [117]. Surgical injury and pain activate sympathetic pathways and increase catecholamines, while the hypothalamic–pituitary–adrenal axis increases cortisol [117,238]. Reviews of the surgical stress response describe consistent endocrine and inflammatory effects that can alter microvascular tone and endothelial function [117,174].

These perioperative changes can matter more in diabetes because baseline vascular control is already impaired [239]. Catecholamine surges can increase arteriolar tone and can narrow capillary segments through mural cell signaling [240,241]. Endothelial oxidative stress can increase during stress responses and can further reduce nitric oxide availability [242,243]. In parallel, endothelial activation can promote leukocyte adhesion and capillary flow disruption [94]. Experimental DR work shows that leukostasis rises early in diabetes and that ICAM-1-linked adhesion contributes to leakage and flow impairment [94].

Perioperative practice can modify several upstream drivers of this physiology. Analgesia and anesthetic technique can reduce pain-related afferent input and can dampen the systemic stress response [117]. Reviews and clinical summaries report that regional anesthesia can reduce postoperative pain and can blunt stress-response physiology in many settings [194]. Perioperative glycemic management is also relevant because hyperglycemia is common during surgical stress and is linked to worse outcomes [244,245]. A meta-analysis relating perioperative glycemic targets to postoperative outcomes supports the clinical importance of avoiding uncontrolled perioperative hyperglycemia in patients with diabetes [246].

Sleep disruption after surgery is another modifiable contributor. Sleep deprivation can shift autonomic balance toward sympathetic predominance and can impair endothelial function in human studies and reviews [116,128,247]. This direction is consistent with the focus of this review because sleep deprivation and postoperative sleep disturbance are linked to endothelial dysfunction and autonomic imbalance with sympathetic predominance, which may together reduce the reliability of activity-evoked retinal blood-flow regulation. This rationale supports perioperative research designs that pair autonomic measures with retinal functional hyperemia testing in patients with diabetes. Perioperative stress may create a short-term risk window in diabetes, but retinal-specific longitudinal data is still limited.

## 9. Future Directions and Testable Predictions

**Figure 5:**
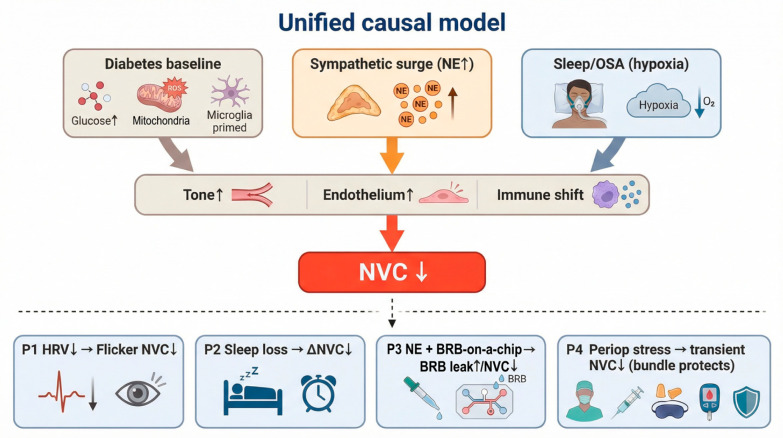
Unified causal model and testable predictions linking diabetes, sympathetic surges, and sleep-related hypoxia to impaired retinal neurovascular coupling. This schematic summarizes how a diabetic baseline lowers retinal vascular reserve and makes neurovascular coupling (NVC) easier to disrupt. The model highlights three upstream drivers: hyperglycemia-associated oxidative stress and microglial priming, acute sympathetic surges with higher norepinephrine (NE), and sleep disturbance/obstructive sleep apnea (OSA) with intermittent hypoxia. These drivers converge on three mechanistic nodes: higher mural cell tone (arterioles/pericytes), impaired endothelial function with reduced nitric oxide (NO) signaling, and a shift toward proinflammatory immune activity. Together, these changes reduce activity-evoked functional hyperemia (NVC), which can worsen local oxygen stress and barrier vulnerability. The lower panels translate this model into testable predictions using existing tools. P1: Lower heart rate variability (HRV) as a marker of higher sympathetic drive will associate with a smaller flicker-evoked NVC response. P2: Acute sleep loss may cause a larger drop in NVC in diabetes than in matched controls. P3: In microphysiological “on-a-chip” systems that model diabetic conditions, NE exposure will increase blood–retinal barrier (BRB) leak and further reduce NVC-related readouts. P4: Perioperative stress may be followed by a transient NVC depression in diabetes, and improved analgesia and supportive perioperative care may mitigate this effect. Arrows indicate the proposed direction of effects or links between components in the unified causal model; Upward arrows indicate an increase, and downward arrows indicate a decrease.

The sympathetic–NVU framework is useful because it produces testable predictions with tools that are already in use. Researchers can quantify retinal NVC with flicker stimulation and DVA [9,248]. Researchers can also quantify stimulus-evoked retinal blood flow with Doppler OCT, which helps separate diameter change from flow change [249,250]. In parallel, researchers can estimate autonomic state with HRV and clinical markers of autonomic dysfunction, which are common in diabetes [251]. Population studies, including large cohorts from China, show that retinal microvascular changes appear early in dysglycemia [252]. These data support the use of functional readouts before advanced fundus signs (Figure 5) [9].

One prediction is that diabetic patients with higher sympathetic drive will show smaller flicker-evoked hyperemia. Prior human work shows that flicker-evoked retinal vasodilation is reduced in type 2 diabetes, and that weaker flicker responses track with future retinopathy outcomes in longitudinal designs [16,22,253]. The recent work also links retinal NVC metrics to HRV in type 2 diabetes, which supports an association between autonomic measures and stimulus-evoked vascular reserve in type 2 diabetes [72]. The available data are cross-sectional, so they do not establish direction or causality. This prediction is easiest to test in a design that measures systemic blood pressure and intraocular pressure, because ocular perfusion pressure depends on both. Researchers can also include Doppler OCT so that they can test whether reduced hyperemia reflects reduced dilation, reduced flow gain, or both [249].

Another prediction is that acute sleep loss will reduce NVC more in diabetes than in matched controls. Sleep restriction increases sympathetic activation and can change catecholamine-related hemodynamics in controlled settings [254]. Sleep deprivation also impairs endothelial function in meta-analytic human evidence, which fits a mechanism that reduces nitric-oxide-linked dilation reserve [116]. A crossover design can compare normal sleep with restricted sleep while researchers measure HRV, standard stress-axis markers, and flicker responses in the same participants. Researchers can then test whether sleep loss produces a larger drop in flicker-evoked dilation or flow in diabetes, and whether the drop tracks with autonomic changes.

A third prediction is that catecholamines will amplify NVU dysfunction under high-glucose or diabetic-like conditions in microphysiological platforms. Recent inner BRB “on-a-chip” systems can reproduce key diabetic retinopathy phenotypes and provide barrier readouts such as permeability and junction organization under controlled flow [213,214]. NVU-on-chip reviews and models also support multicell designs that include endothelium, mural cells, and glial or immune components with time-resolved imaging and pharmacology [255,256]. In these systems, researchers can define norepinephrine as an input and then measure sequential outputs. Researchers can measure pericyte or mural cell calcium and contractile responses, endothelial nitric oxide signaling, barrier permeability, and cytokine release. Researchers can then add an activity trigger and a real-time flow or diameter readout, so the readout remains an NVC endpoint rather than a general injury endpoint. Functional OCTA and related approaches also support this logic by showing that capillary-resolved functional signals can reveal early neurovascular dysfunction in diabetes [23,25]. This prediction tests a defined adrenergic input under controlled conditions, but it does not capture the full neuroendocrine and immune shifts seen with sleep loss or surgery. Therefore, chip findings should be paired with in vivo and human validation.

A fourth prediction is that perioperative sympathetic surges will be followed by transient NVC depression in diabetes, especially when analgesia is inadequate. Surgery activates a neuroendocrine stress response that includes catecholamine and cortisol signaling, and anesthesia choices can modify the magnitude of this response [117,238]. Regional analgesia and neuraxial techniques can reduce stress hormone responses in many settings, which supports the idea that nociceptive blockade can reduce catecholamine exposure to micro vessels [117,194]. Human data on postoperative pain also show that higher pain can be associated with higher plasma norepinephrine, even when the relationship is not perfectly linear across all measures [37]. A practical perioperative study can measure flicker-evoked responses before surgery and after surgery, while researchers track HRV and standard perioperative stress proxies. Researchers can then test whether diabetic participants show a larger postoperative drop in NVC, and whether the drop aligns with stronger sympathetic signatures and higher inflammatory markers.

## 10. Conclusions

Diabetic retinopathy reflects dysfunction across the retinal neurovascular unit, not only capillary injury. Retinal neurovascular coupling is a useful functional marker because it tests whether neural activity still produces an adequate local blood-flow increase. This review argues that sympathetic activation can worsen this process in diabetes. Catecholamines can increase pericyte and arteriolar contractility, oxidative stress can further reduce nitric-oxide-dependent dilation, and barrier stress can increase Müller glia and microglia inflammatory signaling. Together, these changes can reduce vascular reserve and increase variability in activity-evoked perfusion.

Current evidence has clear limits. Only a small number of human studies measure autonomic markers and retinal NVC in the same visit, and many datasets are cross-sectional. Cross-sectional designs can show associations, but they cannot test direction or causality. Autonomic dysfunction may contribute to NVC impairment, but both signals may also reflect shared factors, such as disease duration, baseline blood pressure, medications, glycemic control, and sleep disorders such as OSA. Studies also differ in devices, stimulus settings, and outcome definitions, so comparisons across cohorts remain difficult. Experimental models do not capture full perioperative physiology, and retina-on-a-chip systems still simplify systemic endocrine and immune inputs. These gaps support plausibility, but they do not define causality or effect size across clinical settings.

Even with these limits, the clinical value is direct. Clinicians can measure autonomic state and retinal NVC in the same person, and clinicians can relate both to stress exposures such as sleep loss and perioperative stress. This approach can identify patient subgroups with low autonomic flexibility and low retinal vascular reserve. These subgroups are likely to show larger NVC reductions during stress and may have a higher risk of progression. Future work should prioritize longitudinal designs, standardized flicker and fOCTA protocols, and perioperative studies that pair HRV-based markers with retinal NVC readouts before and after surgery. Future work should also test whether interventions that reduce sympathetic activation, improve sleep, and stabilize perioperative glucose can preserve NVC and limit NVU instability in diabetes.

## Data Availability

No new data were created or analyzed in this study.

## Abbreviations

The following abbreviations are used in this manuscript:

20-HETE	20-hydroxyeicosatetraenoic acid
Ado	Adenosine
AGE–RAGE	Advanced glycation end-product-receptor for AGE
AQP4	Aquaporin-4
ATP	Adenosine triphosphate
BRB	Blood–retinal barrier
cGMP	Cyclic guanosine monophosphate
CO_2_	Carbon dioxide
COX	Cyclooxygenase
COX-2	Cyclooxygenase-2
Cx43	Connexin43
DR	Diabetic retinopathy
DVA	Dynamic vessel analysis
EET	Epoxyeicosatrienoic acid
EETs	Epoxyeicosatrienoic acids
eNOS	Endothelial nitric oxide synthase
fOCTA	Functional optical coherence tomography angiography
GCL	Ganglion cell layer
HbA1c	Glycated hemoglobin (hemoglobin a1c)
HIF	Hypoxia-inducible factor
HIF-1α	Hypoxia-inducible factor-1α
HRV	Heart rate variability
ICAM-1	Intercellular adhesion molecule-1
IL-1β	Interleukin-1 beta
INL	Inner nuclear layer
IOP	Intraocular pressure
iNOS	Inducible nitric oxide synthase
Kir4.1	Inwardly rectifying potassium channel 4.1
NE	Norepinephrine
NE/Epi	Norepinephrine/epinephrine
NLRP3	NLR-family pyrin domain-containing 3
NO	Nitric oxide
nNOS	Neuronal nitric oxide synthase
NVC	Neurovascular coupling
NVU	Neurovascular unit
OCT	Optical coherence tomography
OCTA	Optical coherence tomography angiography
ONL	Outer nuclear layer
OSA	Obstructive sleep apnea
PaCO_2_	Arterial partial pressure of carbon dioxide
PEEP	Positive end-expiratory pressure
PGE2	Prostaglandin E2
PKC	Protein kinase C
ROS	Reactive oxygen species
RPE	Retinal pigment epithelium
sGC	Soluble guanylate cyclase
TEER	Transepithelial electrical resistance
TNF-α	Tumor necrosis factor-alpha
VCAM-1	Vascular cell adhesion molecule-1
VEGF	Vascular endothelial growth factor
ZO-1	Zonula occludens-1
α1	A1-adrenergic receptor
α1B	A1B-adrenergic receptor
β2AR	Β2-adrenergic receptor
